# Pulvinar Sign, Stroke and Their Relationship with Fabry Disease: A Systematic Review and Metanalysis

**DOI:** 10.3390/neurolint14020041

**Published:** 2022-06-01

**Authors:** Juan Fernando Ortíz, María Belén Solís, Syed Saad Ali, Mahika Khurana, Juan Andrés Moncayo, Nishel Yogesh Kothari, Mateo Alzamora, Ahmed Eissa-Garces, Ghanshyam Patel, Gustavo Andrés Monteros, Meghdeep Sen, Jonathan Quiñonez

**Affiliations:** 1 Department of Neurology, California Institute of Behavioral Neurosciences & Psychology, Fairfield, CA 94534, USA; 2 School of Medicine, Colegio de Ciencias de la Salud, Pontificia Universidad Católica del Ecuador, Quito 170083, Ecuador; maria.belen04@hotmail.com (M.B.S.); Jmoncayo725@gmail.com (J.A.M.); gusmonteros@gmail.com (G.A.M.); 3 Department of Internal Medicine, Dow Medical College, Dow University of Health Sciences, Karachi 74200, Pakistan; syedsaada1998@gmail.com; 4 Public Health Department, University of California Berkeley, Berkeley, CA 94720, USA; mahikakhurana@gmail.com; 5 Jawaharlal Nehru Medical College, KLE University, Neuro Nahgar, Belagavi 590010, India; nishelkothari@gmail.com; 6 School of Medicine, Colegio de Ciencias de la Salud, Universidad San Francisco de Quito, Quito 170901, Ecuador; mateoalzamora51@gmail.com (M.A.); aeissag@estud.usfq.edu.ec (A.E.-G.); 7 Mercy Health Internal Medicine Residency, Javon Bea Hospital, Rockford, IL 61114, USA; grp_aaa@yahoo.com; 8 School of Medicine, American University of Antigua, St John’s P.O. Box W1451, Antigua and Barbuda; meghdeepsen@gmail.com; 9 Department of Osteopathic Neuromuscular Medicine, Larkin Community Hospital Palm Springs Campus, Hialeah, FL 33012, USA; jquinonez@larkinhospital.com

**Keywords:** stroke, Fabry, pulvinar, renal failure, metanalysis

## Abstract

Background: Fabry disease (FD) is the second most common lysosomal storage disorder. This disorder affects multiple systems that include the cardiac, renal, and nervous system. The pulvinar sign (PS) is a relatively common sign seen in patients with FD. The PS is a bilateral, symmetrical pulvinar high signal relative to the signal intensity seen on unenhanced T1-weighted brain MR imaging. Methods: We conducted a systematic review with metanalysis to analyze the pool prevalence of the disorder. We used the Moose Guidelines and PRISMA Protocol for this systematic review and Robins 1 to access the BIAS of the study. To analyze the pool prevalence, we used “Open Meta-Analysis” software for analyzing the study. We used “Review Manager 5.4” to analyze the odds ratio between patients with and without the PS and patients with and without stroke among patients with FD. Results: We gather 12 studies from 2003 to 2021 for the analysis of this study. The pool prevalence of the study was 0.146 (0.076–0.217) (62/385 cases) with a 95% CI (0.0945–0.415) (*p* < 0.01). The prevalence was much higher in men (59 cases) than in women (3 cases). There was no relationship between the pulvinar sign and patients with stroke among patients with Fabry disease. Odds ratio 1.97 95% CI (0.35–11.21), *p* = 0.44; Tau2 = 0.77. There seems to be a correlation with renal failure (RF), but there were very few studies to conduct a metanalysis with RF. Conclusions: The prevalence of the PS among all studies was 23.9%; the prevalence of this sign is higher among males. We found that FD patients who had strokes did not have higher odds of presenting with the Pulvinar Sign than the FD patients who did not suffer a stroke. Patients with renal failure and FD seem to have a higher tendency to have the PS, but there were not enough studies to analyze that theory. Overall, we think the pulvinar sign has a poor prognostic value in patients with Fabry’s disease.

## 1. Introduction

Fabry disease (FD) is a rare X-linked lysosomal disorder caused by the enzyme alpha-galactosidase A’s low or absent activity, resulting in the accumulation of GL-3 globotriaosylceramide and neutral glycosphingolipids [1]. This underdiagnosed condition has no racial or ethnic distinction. Its prevalence is estimated at 1:17,000–1:117,000. The prevalence of the classic FD phenotype is between 1:22,000–1:40,000 in males, whereas the late-onset FD phenotype is associated with a male prevalence between 1:1000–1:3000 and 1:6000–1:40,000 in females [2]. Severity of FD depends on sex: males present a more severe form of the disease as opposed to females [3].

Glycosphingolipid deposition starts in lysosomes and ultimately causes organ failure. This deposition is predominantly in the endothelium, mainly in the media of small vessels, cardiac muscle, renal tubules, autonomic ganglia, conducting fibers, and cortical and brain stem structures. These findings exemplify the clinical manifestations of FD; the most important are cardiomyopathy, renal failure, and cerebrovascular accidents (CVAs), inducing premature death [4].

The disease usually manifests in childhood and adolescence with painful acroparesthesias, hypohidrosis, gastrointestinal symptoms, cutaneous manifestations that can include umbilical petechiae and lenticular opacities [1]. It generally manifests with proteinuria and albuminuria, which can progress to renal failure [5]. Nervous system involvement commonly presents with white matter lesions or vascular involvement such as stroke, transient ischemic attack, and vessel ectasia of posterior circulation [1,6]. These abnormalities are seen on MRI as white matter hyperintensities and increased basilar artery diameter [6]. The prevalence of ischemic stroke or transient ischemic attack for patients with FD is about 12.2 times greater than expected in a comparable general population [7]. Stroke in these patients occurred in 6.9% of males and 4.3% of females [7]. 

Important MRI findings in Fabry disease are dolichoectatic vertebrobasilar vessels, brain atrophy, and periventricular white matter intensities [8,9]. Another important finding in FD is the PS, which is an atypical hyperintensity in the thalamus on T1-weighted images called pulvinar sign (PS) [10]. The PS has also been described as a hyperintensity in the FLAIR, DWI MRI sequence in other conditions such variant Creutzfeldt-Jakob, Anti Hu, and anti-CV2 encephalitis [11,12,13].

The PS exclusively involves the lateral thalamic pulvinar with symmetric bilateral hyperintensity on FLAIR images, which are important nuclei for visual processing [14]. It has been considered a common radiological sign of FD without being pathognomonic. 

To the best of our knowledge, there has not been a systematic review or a metanalysis of PSs and their presence in the FD. We present this meta-analysis aiming to calculate the pool prevalence among multiple studies and seek to describe and characterize the relationship between FD PS and its association with stroke and renal failure.

## 2. Materials and Methods

### 2.1. Protocol

This systematic review was conducted by following the Meta-Analysis of Observational Studies in Epidemiology (MOOSE) and PRISMA protocol reporting guidelines [15]. 

### 2.2. Eligibility Criteria and Study Selection

Inclusion Criteria: Only observational studies conducted among human subjects in the last 18 years were considered, as the PS was reported for the first time in 2003 as pulvinar hyperintensities on T1 MRI sequence. 

Exclusion Criteria: We exclude systematic reviews, metanalyses, and cases reports. Additionally, we exclude articles that did not mention the prevalence of the PS among patients with FD. The PS or pulvinar intensity on T1 sequence was first described in 2003, which is why we excluded articles before this year. 

### 2.3. Database and Search Strategy 

For this systematic review, we used PubMed as a database. The search was performed between 25 October 2021 and 25 January 2021. (“pulvinar sign” [Title/Abstract] AND “fabry disease” [Title/Abstract]) OR (“Pulvinar” [Title/Abstract] AND “fabry disease” [Title/Abstract]) OR ((“Pulvinar” [MeSH Terms] OR “Pulvinar” [All Fields] OR “pulvinars” [All Fields]) AND “Hyperintensities” [Title/Abstract] AND “fabry disease” [Title/Abstract]) OR (“fabry disease” [Title/Abstract] AND “MRI” [Title/Abstract]) OR (“fabry disease” [Title/Abstract] AND “Stroke” [Title/Abstract]) OR (“fabry disease” [Title/Abstract] AND “renal disease” [Title/Abstract]).

### 2.4. Data Extraction 

The following information was collected: author, year of publication, country of publication, study type, methodology, outcomes, prevalence of pulvinar sign in patients with FD, and the prevalence of patients with stroke, acute renal failure, and FD. 

Two individuals (M.B.S. & J.F.O.) extracted the data independently. 

### 2.5. Bias Analysis

To assess and minimize bias, the risk of bias in non-randomized studies (ROBINS-I) tool was used for observational studies [16]. 

### 2.6. Data Analysis 

For the analysis of the data, we used two software programs; for the prevalence of the pulvinar sign, we used “Open Meta-Analysis,” and to analyze the relation of stroke and the pulvinar sign, we used “Review Manager 5.4.”

## 3. Results

**Figure 1:**
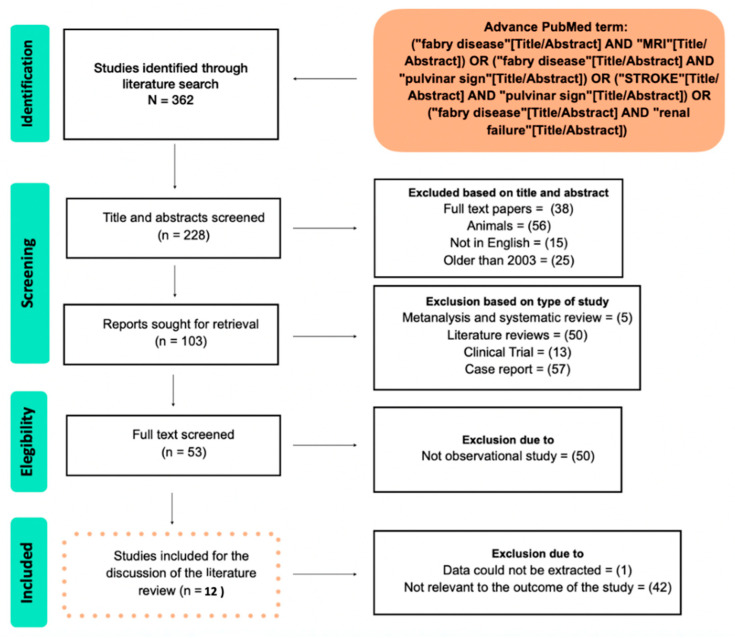
Flowchart of the study.

Figure 1 shows the PRISMA flow chart of the metanalysis.

The characteristics of the studies of the metanalysis are shown in Table 1 [6,10,17,18,19,20,21,22,23,24,25,26].

**Figure 2:**
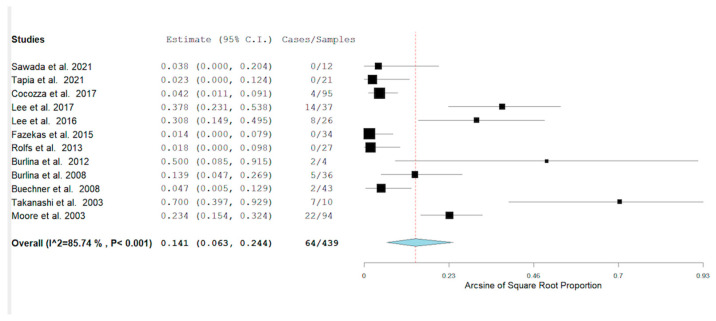
The pool prevalence of the 12 collected studies was 64/439, with a pool prevalence of 0.141 *p* ≤ 0.001. 95% CI (0.063–0.244).

We collected 12 studies across the metanalysis. Figure 2 shows the metanalysis of the prevalence of the PS among patients with FD [10,14,17,18,19,20,21,22,23,24]. Figure 2 also shows the pool prevalence among the included studies [10,14,17,18,19,20,21,22,23,24,25,26].

The highest rate of the PS was found in Takanashi et al. (70%), while the rate of the PS was 0 in 4 studies [17,19,24,26]. Table 2 documents the presence or not of stroke among patients with Fabry disease with/without the PS [10,17,21,23,24].

**Figure 3:**
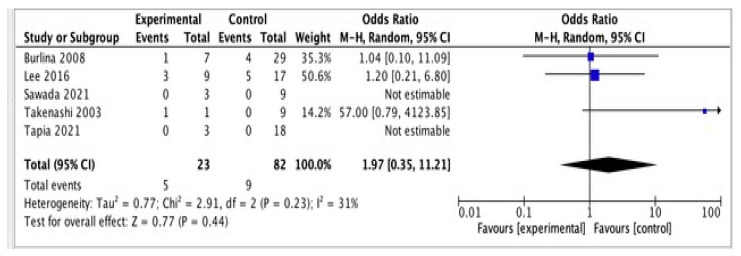
Shows the relationship between pulvinar sign and stroke.

Five studies compared the prevalence of stroke in patients with and without the PS. The analysis is shown in Figure 3 [10,17,23,24].

5/23 patients had a stroke and the pulvinar sign vs. 9/82 who had the pulvinar sign but no related stroke. Patients with the PS do not have higher odds of having a stroke 1.97 (0.35, 11,21) 95 CI% (*p* = 0.44).

We also analyzed and measured the frequency of renal failure in patients with and without PS among patients with PS [6,10]. Table 3 shows the analysis.

There, 8/9 patients with renal failure have the pulvinar sign among 169 patients with FD across two studies [6,10]. They were not enough studies to conduct a metanalysis and explore the relationship between RF and the PS.

## 4. Discussion

### 4.1. The Pulvinar, the Pulvinar Sign, and Pathophisiology

The pulvinar nucleus (PN) is the largest nucleus of the thalamus and plays a role in visual attention and modification of behavior response [27]. Their main connections are the superior colliculus and regions of the dorsal visual stream projecting to the posterior parietal cortex [27]. The PN accounts for 30% of the volume of the thalamus and is supplied by the posterior choroidal artery [27]. Patients with lesions in the PN have transitory deficits in encoding the contralateral visual field. They also have difficulty localizing stimuli on the contralateral visual field [27].

The PS has also been described in neurological disorders of different etiologies, suggesting their sensitivity of the PN. The diseases that have reported the PS included: Tay Sachs, Krabbe disease, CNS Infections, radiation [6], variant Creutzfeldt-Jakob disease [11], anti CV2 encephalitis [11], Wernicke encephalopathy [28], Anti HU encephalitis [13], limbic encephalitis [29], neurosarcoidosis [30], and Post Influenza Encephalitis [31].

The PS is defined as a bilateral, symmetrical pulvinar high signal relative to the signal intensity of other deep grey matter nuclei and cortical grey matter on unenhanced T1-weighted brain, FLAIR, or DWI MRI imaging [10]. The pathognomonic role of the PS is mainly unknown. Various substances increase the intensity of T1 imaging such as fat, calcium, manganese, iron, melanin, free radicals, and elevated protein [20]. When Fat Suppression is performed, the pulvinar sign is not visualized, ruling out fat deposition as a cause of the PS [20].

The PS can be attributed to tissue mineralization [20]. Specifically, calcium deposition presents as hyperdensity on CT scans. On MR imaging, the T1 shortening effect is due to the interaction of calcified tissue and protons. At a lower calcium concentration below 30–40%, hyperintensity is seen in T1 weighted images. However, when there is an increasing concentration above 30–40%, the hyperintensity disappears [20].

The PS has been found in multiple metabolic disorders [10]. The PS is likely caused by dystrophic calcification which can be seen on CT scans [6] Moreover, calcifications caused by deposits of calcium and mineralization with other metals such as zinc, magnesium, and chromium can contribute to the sign [20]. Vascular abnormalities are also related [6]. The PS can indirectly indicate disturbance of the posterior circulation. However, the mechanism is uncertain [32]. Microvascular alterations might also be related to the sign [6]. 

### 4.2. Prevalence of the Pulvinar Sign in Fabry Disease

Twelve studies have examined the incidence of the PS. There is a significant discrepancy in the prevalence of the PS in FD. Sawada et al., Fazekas et al., and Rolfs and Tapia et al. reported no prevalence of the pulvinar sign in patients with FD [17,19,24]. In addition, we found studies, such as Moore, Lee, and Takanashi, which reported high prevalences of 23.4, 32.4, and 70% [18,20,21,23]. The study conducted by Moore et al. was only conducted in male patients, where a higher prevalence was reported in comparison to females [6,20]. The sample size in the study conducted by Takanashi et al. was too small, which could explain the high rate of the PS [22]. 

The PS in FD seems to be influenced by phenotype. To our current knowledge, there are two clinical phenotypes of Fabry disease: classical and later-onset (non-classical) phenotypes. In the non-classical phenotype, renal, cardiac, and/or cerebrovascular manifestations are seen in adulthood [33]. Within the later-onset form, there is also a particular late-onset phenotype with a predominant cardiac variant, an intronic variant (IVS4 + 919G > C), common in Asian countries [23].

According to our study, in the classical phenotype, the prevalence of the PS seems to be higher than in the non-classical. A direct comparison of phenotypes is appreciated in Lee et al.’s study; in patients with late-onset Fabry disease, the frequency of the pulvinar sign was 8/32 (25%) vs. 6/12 (50%) [18]. Rolfs and Fazekas only include patients with non-classical late-onset variants of Fabry disease, and the prevalence in those studies was zero [12,21]. It is fair to point out that the prevalence of zero was not exclusive to the non-classical variant. In Tapia et al. and Sawada et al.’s studies, the prevalence was zero as well [17,24]. While overall the PS seems to be more prevalent in the classical phenotype, this is not entirely true for a particular late-onset phenotype with a predominant cardiac variant (IVS4 + 919G > C). Lee’s study only included patients with one type of non-classical variant, the cardiac Fabry’s phenotype, and the prevalence was high at 32.4% [23]. 

The prevalence of the PS in women was significantly less when compared to men. Burlina et al. and Lee et al. reported two and one female patients, respectively, with the PS [21,23]. The PS was faint compared to what was reported in male patients according to Burlina et al. [21]. The study’s author suggested that this could be related to the younger age of the patients, meaning they could develop the PS as they age [21]. Females have a residual level of the enzyme, meaning that the residual enzyme could have a protective effect on developing the pulvinar sign [10].

There are also marked differences regarding age when comparing the classical and the non-classical phenotype of FD. Lee et al. found that subjects with the classical variant had a mean age of nine years old, in contrast to the non-classical variant (50 years old). There was an increased occurrence of the PS in the classical phenotype, but it was not significant (50% vs. 32%, respectively, with a *p*-value of = 0.4701). However, the sample was small [18].

### 4.3. Pulvinar Sign and Correlation with Disease 

#### 4.3.1. Stroke

Previous studies have suggested that the pulvinar sign has poor prognostic value and utility in patients with FD [6]. It has been estimated that, during the course of the disease, 16% of patients will experience a stroke [34]. According to this study, the presence of the pulvinar sign in FD patients does not correlate with the possibility of having a stroke. In six studies, the overall odds ratio for stroke in FD patients with pulvinar sign was 1.97 (0.35–11.21). The present study has demonstrated no significant association between stroke and the pulvinar sign in FD patients; however, additional longitudinal studies are necessary to establish a relationship between these two conditions.

#### 4.3.2. Renal Failure

Two studies (Burlina et al. and Cocozza et al.) showed a high prevalence of a renal disease among male patients [6,10]. We could not perform a metanalysis on patients with renal failure because there were only two studies to compare. Other studies documented patients with renal involvement but did not specify if the involvements were simple proteinuria, glomerular disease, or renal failure, so we decided not to further analyze this because of lower statistical power to see a significant difference. 

An explanation for the increased number of cases in patients with renal disease could be related to the deposition of gadolinium in this structure [6]. Recent evidence suggests that multiple contrast infusions could accumulate contrast derivatives in deep gray matter structures, including the pulvinar. However, hyperintensity was not observed in the dentate nucleus, the place where gadolinium deposits the most, dissipating this theory. Additionally, contrast is not usually given to patients with RF [6]. The degree of kidney damage did not seem to be associated with the PS [10]. 

However, patients with poor renal dysfunction could have decreased clearance of metals or other substances that may accumulate in deep gray matter structures such as the pulvinar [6]. In the early studies, the pulvinar sign could have been considered a pathognomonic sign. However, different studies have documented this sign in different conditions [20]. Because it is not unique to Fabry disease, it should not be considered a pathognomonic sign.

### 4.4. Limitations

The heterogenicity of the publications was a significant limiting factor for the study. For example, Moore et al. found a high prevalence of the pulvinar sign, but the paper only included male patients in their cohort [20]. Regarding the detection of pulvinar signs in female Fabry patients, we should mention the report of Burlina et al. (2012), where two sisters out of four showed PSs on brain MRI.

Additionally, this metanalysis includes studies where only a specific phenotype of FD was used (Lee’s, Rolf’s, and Fazekas’ studies) [18,19,26]. As mentioned in the discussion, the prevalence of the PS seems to vary among different phenotypes. So, further studies specifying the prevalence of the PS in each phenotype might be beneficial. 

There were not enough studies to analyze the relation between renal failure and PS, and more studies need to be conducted to explore this relationship.

## 5. Conclusions

The prevalence of the PS varies across the studies, and the pulvinar sign is more frequently seen among men than women. The disease seems to be correlated with acute renal failure, but there were not enough studies to analyze this association. There is no association between the pulvinar sign and the development of stroke among patients with Fabry disease.

Among phenotypes, the sign is more frequent in the classical phenotype than the non-classical phenotype; among the non-classical phenotypes, the cardiac variant has a higher prevalence of the pulvinar sign. Nevertheless, only the Asian cardiac variant was analyzed.

## Figures and Tables

**Table 1:** The characteristics of the studies included in the metanalysis.

Author, Year, Country	Study Type	Sample Size	Age (Years)	Prevalence of the Pulvinar Sign
Sawada, 2021, Japan [17]	Cross-sectional—Single-center	10 Males	53.8	0
		2 Females		
Cocozza et al., 2017, Italy [6]	Cross-sectional— Multicenter study	80 Males	41 ± 13.8	4
		53 Females		
Lee et al., 2017, Taiwan [18]	Cross-sectional study	23 Males	53.9 ± 7.2	12
		14 Females		
Fazekas et al., 2015, Europe [19]	Cross-sectional study	Females		0
		Males		
Moore et al., 2003, USA [20]	Cross-sectional study	0 Memale	35 ± 12	22
		94 Males		
Burlina et al., 2008, France and Italy [10]	Cross-sectional study	16 Males	40	5
		20 Females		
Takanashi et al., 2003, Japan [22]	Cross-sectional study	9 Males	36.9	7
		1 Female		
Burlina, 2012, Italy, Argentina and France [21]	Case Series	4 Females	25.25	2
Lee et al., 2016, Taiwan [23]	Cross-sectional study	20 Males	59.5 ± 7.2	8
		6 Females		
Tapia et al., 2021, USA [24]	Cross-sectional study	8 Males	50 ± 13.4	0
		13 Females		
Buechner et al., 2008, Italy [25]	Cross-sectional study	25 males	41.94 ± 10.83	0
		18 Females	52.48 ± 17.50	2
Rolfs, Europe [26]	Crossectional -Multicenter Study	16 Females 11 Males	Not Reported	0

**Table 2:** Shows the frequency of stroke in patients with Fabry disease with/without PS—Pulvinar Sign and WPS—Without Pulvinar Sign.

Autor, Year	Stroke	Without Stroke
Burlina, 2008 [10]	1 PS	4 PS
	6 WPS	25 WPS
Lee, 2016 [23]	3 PS	5 PS
	6 WPS	12 WPS
Sawada et al., 2021 [17]	0 PS	0 PS
	3 WPS	9 WPS
Tapia et al., 2021 [24]	0 PS	0 PS
	3 WPS	18 WPS
Takenashi et al., 2003 [22]	1 PS	0 PS
	0 WPS	9 WPS

**Table 3:** Shows the frequency of renal failure in patients with Fabry Disease. PS—Pulvinar Sign and WPS—Without Pulvinar Sign [6,25].

Autor, Year	Renal Failure	Without Renal Failure
Burlina, 2008 [10]	4 PS	1 PS
	1 WPS	30 WPS
Cocozza, 2017 [14]	4 PS	0 PS
	39 WPS	90 WPS

## Data Availability

Not applicable.

## References

[1] Bokhari S.R.A., Zulfiqar H., Hariz A. (2022). Fabry Disease. StatPearls.

[2] Fabry Disease. https://rarediseases.org/rare-diseases/fabry-disease/#:~:text=Fabry%20disease%20is%20a%20rare,known%20as%20lysosomal%20storage%20disorders..

[3] Chan B., Adam D.N. (2018). A Review of Fabry Disease. Ski. Ther. Lett..

[4] Michaud M., Mauhin W., Belmatoug N., Garnotel R., Bedreddine N., Catros F., Ancellin S., Lidove O., Gaches F. (2020). When and How to Diagnose Fabry Disease in Clinical Pratice. Am. J. Med. Sci..

[5] Schiffmann R., Waldek S., Benigni A., Auray-Blais C. (2010). Biomarkers of Fabry Disease Nephropathy. CJASN.

[6] Cocozza S., Russo C., Pisani A., Olivo G., Riccio E., Cervo A., Pontillo G., Feriozzi S., Veroux M., Battaglia Y. (2017). Redefining the Pulvinar Sign in Fabry Disease. AJNR Am. J. Neuroradiol..

[7] Kolodny E., Fellgiebel A., Hilz M.J., Sims K., Caruso P., Phan T.G., Politei J., Manara R., Burlina A. (2015). Cerebrovascular involvement in Fabry disease: Current status of knowledge. Stroke.

[8] Crutchfield K.E., Patronas N.J., Dambrosia J.M., Frei K.P., Banerjee T.K., Barton N.W., Schiffmann R. (1998). Quantitative analysis of cerebral vasculopathy in patients with Fabry disease. Neurology.

[9] Ginsberg L., Manara R., Valentine A.R., Kendall B., Burlina A.P. (2006). Magnetic resonance imaging changes in Fabry disease. Acta Paediatr. Suppl..

[10] Burlina A.P., Manara R., Caillaud C., Laissy J.-P., Severino M., Klein I., Burlina A., Lidove O. (2008). The pulvinar sign: Frequency and clinical correlations in Fabry disease. J. Neurol..

[11] Fadda L., Floris G., Polizzi L., Meleddu L., Ercoli T., Garofalo P., Saba L., Muroni A., Defazio G. (2018). Pulvinar sign in a case of anti-CV2 encephalitis. J. Neurol. Sci..

[12] Brandel J.-P., Knight R. (2018). Variant Creutzfeldt-Jakob disease. Handb. Clin. Neurol..

[13] Gamache P.-L., Gagnon M.-M., Savard M., Émond F. (2016). Pulvinar sign in a case of anti-HU paraneoplastic encephalitis. Neuroradiol. J..

[14] Cocozza S., Russo C., Pontillo G., Pisani A., Brunetti A. (2018). Neuroimaging in Fabry disease: Current knowledge and future directions. Insights Imaging.

[15] MOOSE Reporting Guidelines for Meta-analyses of Observational Studies|Guidelines|JAMA Surgery|JAMA Network. https://jamanetwork.com/journals/jamasurgery/article-abstract/2778476.

[16] Sterne J.A., Hernán M.A., Reeves B.C., Savović J., Berkman N.D., Viswanathan M., Henry D., Altman D.G., Ansari M.T., Boutron I. (2016). ROBINS-I: A tool for assessing risk of bias in non-randomised studies of interventions. BMJ.

[17] Sawada J., Nakagawa N., Kano K., Saito T., Katayama T., Sawada T., Momosaki K., Nakamura K., Hasebe N. (2021). Characteristics of Neurological Symptoms in Adult Japanese Patients with Fabry Disease. Intern. Med..

[18] Lee H.-J., Hsu T.-R., Hung S.-C., Yu W.-C., Chu T.-H., Yang C.-F., Bizjajeva S., Tiu C.-M., Niu D.-M. (2017). A comparison of central nervous system involvement in patients with classical Fabry disease or the later-onset subtype with the IVS4 + 919G > A mutation. BMC Neurol..

[19] Fazekas F., Enzinger C., Schmidt R., Grittner U., Giese A.-K., Hennerici M.G., Huber R., Jungehulsing G.J., Kaps M., Kessler C. (2015). Brain magnetic resonance imaging findings fail to suspect Fabry disease in young patients with an acute cerebrovascular event. Stroke.

[20] Moore D.F., Ye F., Schiffmann R., Butman J.A. (2003). Increased signal intensity in the pulvinar on T1-weighted images: A pathognomonic MR imaging sign of Fabry disease. AJNR Am. J. Neuroradiol..

[21] Takanashi J., Barkovich A.J., Dillon W.P., Sherr E.H., Hart K.A., Packman S. (2003). T1 hyperintensity in the pulvinar: Key imaging feature for diagnosis of Fabry disease. AJNR Am. J. Neuroradiol..

[22] Burlina A.P., Politei J., Cinque S., Soliani A., Carlier R.Y., Germain D.P., Manara R. (2012). The pulvinar sign in Fabry patients: The first report in female patients. J. Neurol..

[23] Lee H.-J., Hung S.-C., Hsu T.-R., Ko S.-C., Chui-Mei T., Huang C.-C., Niu D.-M., Lin C.-P. (2016). Brain MR Imaging Findings of Cardiac-Type Fabry Disease with an IVS4+919G>A Mutation. AJNR Am. J. Neuroradiol..

[24] Tapia D., Floriolli D., Han E., Lee G., Paganini-Hill A., Wang S., Zandihaghighi S., Kimonis V., Fisher M. (2021). Prevalence of cerebral small vessel disease in a Fabry disease cohort. Mol. Genet. Metab. Rep..

[25] Buechner S., Moretti M., Burlina A.P., Cei G., Manara R., Ricci R., Mignani R., Parini R., Di Vito R., Giordano G.P. (2008). Central nervous system involvement in Anderson-Fabry disease: A clinical and MRI retrospective study. J. Neurol. Neurosurg. Psychiatry.

[26] Rolfs A., Fazekas F., Grittner U., Dichgans M., Martus P., Holzhausen M., Böttcher T., Heuschmann P.U., Tatlisumak T., Tanislav C. (2013). Acute cerebrovascular disease in the young: The Stroke in Young Fabry Patients study. Stroke.

[27] Benarroch E.E. (2015). Pulvinar: Associative role in cortical function and clinical correlations. Neurology.

[28] Khan F., Sharma N., Ud Din M., Bansal V. (2020). Isolated Pulvinar/Hockey Stick Sign in Nonalcoholic Wernicke’s Encephalopathy. Am. J. Case Rep..

[29] Mihara M., Sugase S., Konaka K., Sugai F., Sato T., Yamamoto Y., Hirota S., Sakai K., Sakoda S. (2005). The “pulvinar sign” in a case of paraneoplastic limbic encephalitis associated with non-Hodgkin’s lymphoma. J. Neurol. Neurosurg. Psychiatry.

[30] Wilke M., Dechent P., Bähr M. (2017). Sarcoidosis Manifestion Centered on the Thalamic Pulvinar Leading to Persistent Astasia. Mov. Disord. Clin. Pract..

[31] Tomás J., Macário M.C., Gaspar E., Santana I. (2015). Severe post-influenza (H1N1) encephalitis involving pulvinar nuclei in an adult patient. BMJ Case Rep..

[32] Üçeyler N., Homola G.A., González H.G., Kramer D., Wanner C., Weidemann F., Solymosi L., Sommer C. (2014). Increased Arterial Diameters in the Posterior Cerebral Circulation in Men with Fabry Disease. PLoS ONE.

[33] Azevedo O., Cordeiro F., Gago M.F., Miltenberger-Miltenyi G., Ferreira C., Sousa N., Cunha D. (2021). Fabry Disease and the Heart: A Comprehensive Review. Int. J. Mol. Sci..

[34] Viana-Baptista M. (2012). Stroke and Fabry disease. J. Neurol..

